# Endoscopic Third Ventriculostomy Using Penumbra Artemis™ Neuro Evacuation Device: Technical Case Report and Operative Video

**DOI:** 10.7759/cureus.45761

**Published:** 2023-09-22

**Authors:** Daniel G Lynch, Shyle H Mehta, Kevin A Shah, Daniel Toscano, Rachel Pruitt, Henry H Woo

**Affiliations:** 1 Neurological Surgery, Northwell Health, Manhasset, USA; 2 Pediatric Neurosurgery, Johns Hopkins University, Baltimore, USA

**Keywords:** artemis neuro evacuation device, high-riding basilar artery, intracranial metastasis, obstructive hydrocephalus, endoscopic third ventriculostomy

## Abstract

Endoscopic third ventriculostomy (ETV) is an effective cerebrospinal fluid diversion procedure but can be complicated by the presence of a high-riding basilar artery (BA). A 70-year-old female presented with obstructive hydrocephalus caused by melanoma metastatic to the brainstem in the setting of a high-riding BA. ETV was successfully performed using the Penumbra Artemis™ Neuro evacuation device (Artemis; Penumbra Inc, Alameda, CA, USA) to minimize the risk of injury to the BA. This is to our knowledge the first known Artemis-assisted ETV reported in the English literature, which may reduce the risk of BA injury in selected patients. Further characterization of the benefits and limitations of this procedure is needed.

## Introduction

Hydrocephalus is a common neurosurgical condition caused by alterations in cerebrospinal fluid (CSF) production, flow, and/or reabsorption that can result in permanent neurological impairment [1]. Obstructive hydrocephalus occurs due to congenital or acquired blockage of normal CSF drainage, commonly caused by congenital birth defects in pediatric populations as well as intraventricular hemorrhage and intracranial metastases in adults [2]. While CSF diversion techniques such as ventriculoperitoneal shunting (VPS) have been widely used in the treatment of obstructive hydrocephalus, they have a significant rate of serious complications and long-term shunt malfunction. For example, some studies report a greater than 80% complication and revision rate [3]. Endoscopic third ventriculostomy (ETV), a procedure in which the floor of the third ventricle is fenestrated to allow CSF drainage into the subarachnoid space, has been demonstrated as an effective alternative technique for CSF diversion. In adult populations, ETV is a safe and effective treatment for hydrocephalus, with reported long-term success rates greater than 70% and a 4% rate of complications [4]. Major complications of ETV in the intraoperative period include BA injury due to the proximity of the BA apex to the tuber cinereum, which, while rare (occurring in less than 1% of ETV procedures), is potentially devastating [5]. In the postoperative period, the chief concern is for stoma closure and recurrence of hydrocephalus. The risk for stoma closure and ETV failure is initially higher than the rate of VPS failure, but after three months the risk for failure is equal between the procedures, and at two years ETV is twice as likely to be successful [6]. While ETV generally has high success rates in well-selected patients, there are anatomic considerations that affect the likelihood of procedural success. A high-riding basilar artery (BA) is an anatomic variant in which the bifurcation of the BA lies immediately inferior to the floor of the third ventricle, which increases the risk for BA damage and presents an anatomic challenge to ETV [7], requiring modifications to standard ETV technique. Here the authors present a novel use of the Artemis™ Neuro evacuation device (Artemis; Penumbra Inc, Alameda, CA, USA) to perform endoscopic fenestration of the third ventricular floor in the setting of high-riding BA.

## Case presentation

Clinical presentation

**Figure 1:**
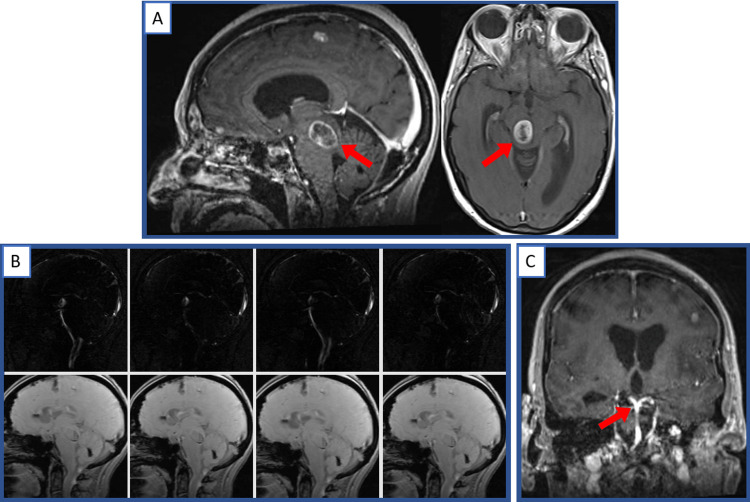
Preoperative imaging MRI with contrast demonstrating a 2cm ring-enhancing mass in the posterior right pons (arrows) compressing the cerebral aqueduct with moderate enlargement of the lateral ventricles visible on axial views (A). MRI flow study (B) showed no flow through the cerebral aqueduct. Additionally, the apex of the basilar artery (arrow) was in close proximity to the floor of the third ventricle (C). This figure was prepared for this submission using original images, and no credit/license is required.

A 70-year-old female with a history of melanoma with intracranial metastases was brought to the emergency department by family members for subacute onset of weakness, fatigue, and diplopia. Their oncologic history was notable for stage IV melanoma on immunotherapy (pembrolizumab, ipilimumab, and nivolumab) with known metastases to the brain for which she had recently begun stereotactic radiosurgery (SRS). Her primary oncologic management at an outside hospital. She had been experiencing three weeks of worsening subjective left-sided upper/lower extremity weakness and short-term memory loss and began to develop blurry vision and diplopia, causing her to present for evaluation. Her exam was significant for a partial right oculomotor nerve palsy, mild (4+/5) left upper and lower extremity weakness, left-sided ataxia, and slight left upper extremity pronator drift. CT without contrast was performed, showing multiple intracranial metastases with associated vasogenic edema, including a 1.8cm mass in the posterior brainstem at the level of the cerebral aqueduct with obstructive hydrocephalus. MRI of the brain with and without contrast was performed revealing a ring-enhancing mass in the right posterior pons (Figure 1A) compressing the cerebral aqueduct with moderate associated hydrocephalus but no midline shift, as well as multiple metastatic lesions in the bilateral frontal and temporal regions. MRI flow study was performed (Figure 1B) demonstrating a lack of flow through the aqueduct. Preoperative imaging also demonstrated a high-riding BA, with approximately 2.5mm separating the apex of the BA and the floor of the third ventricle (Figure 1C). She was admitted to the neurosurgical intensive care unit for hydrocephalus and concern for herniation. A decision was made to perform ETV to relieve obstructive hydrocephalus, however, the presence of a high-riding BA made the procedure more technically challenging.

**Video 1:** Operative video Operative video demonstrating the use of the Artemis neuro evacuation device in ETV. The patient’s presentation and preoperative imaging are briefly reviewed, demonstrating acute obstructive hydrocephalus secondary to a brainstem metastasis compressing the cerebral aqueduct. The basilar artery apex was also high, less than 2.5mm from the floor of the third ventricle. ETV was performed, with the neuroendoscope advanced from the right lateral ventricle to the third ventricle without damaging the septal or thalamostriate veins. The floor of the third ventricle was visualized and the Artemis device was used to elevate and fenestrate the third ventricular floor without damaging the basilar artery. The neruoendoscope was then withdrawn without significant bleeding. The patient tolerated the procedure well with flow into the subarachnoid space and reduction in hydrocephalus on postoperative imaging.

Operative procedure

**Figure 2:**
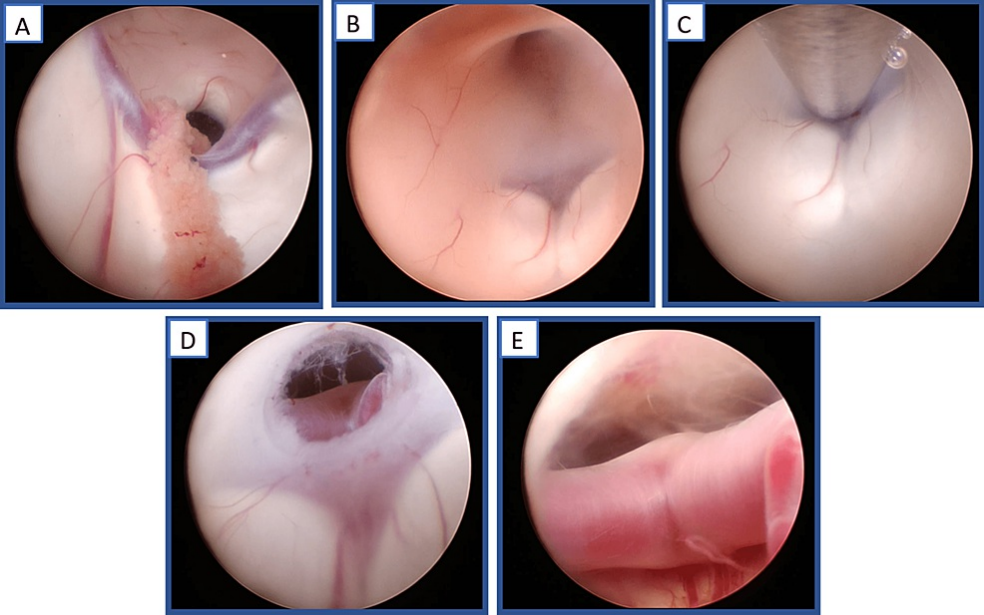
Representative operative images After entry of the neruoendoscope into the lateral ventricle, the choroid plexus, septal vein medially, thalamostriate vein laterally, and foramen of Monro were observed (A). After entry into the third ventricle, the floor of the third ventricle along with the mammillary bodies, optic chiasm, and infundibular recess was visible (B). Using gentle suction, the floor of the third ventricle was elevated (C) to decrease the risk of basilar artery injury during fenestration (D). The basilar artery was inspected following fenestration and found to be undamaged (E). This figure was prepared for this submission using original images, and no credit/license is required.

Under general anesthesia, the patient was prepared, draped, and registered with intraoperative navigation systems. A linear incision was made lateral of the midline on the right side in the anterior-posterior direction. Using a high-speed drill, a single burr hole was made after which the underlying dura was coagulated, a cruciate incision was made, and the dural leaflets were coagulated exposing the pia mater. Thwas e pia mater was opened and under navigation guidance, a peel-away sheath inserted into the right lateral ventricle. The inner stylet was removed, the peel-away sheath anchored to the scalp and the neuroendoscope inserted with visualization of the choroid plexus, septal vein medially, thalamostriate vein laterally, and foramen of Monro (Figure 2A).

**Figure 3:**
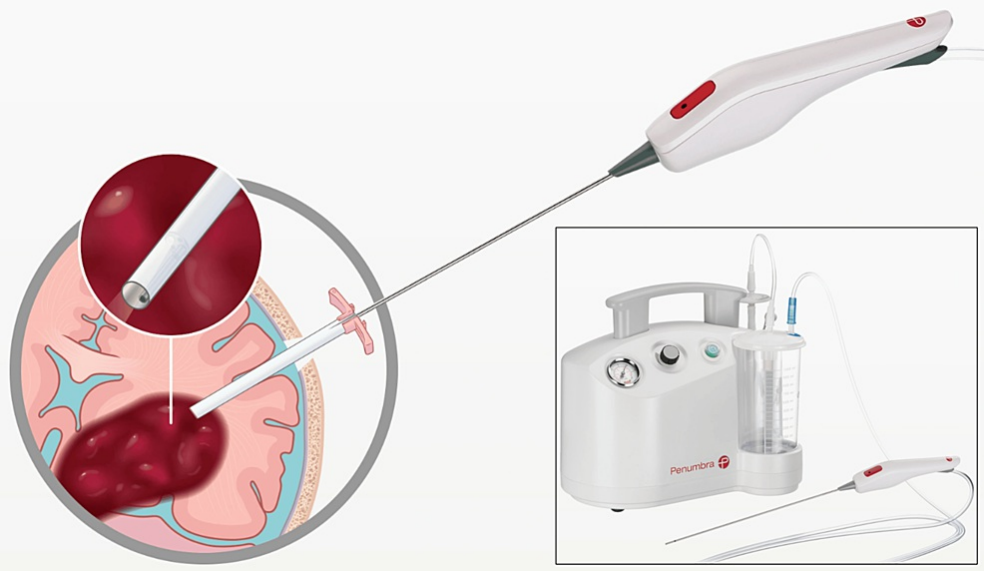
Artemis neuro evacuation device and system The Artemis neuro evacuation device may be used with a neuroendoscope via a 19F peel-away sheath to evacuate tissue and/or fluid from the ventricles or cerebrum, such as in minimally invasive evacuation of intraparenchymal hemorrhage (pictured). The Artemis suction system (insert) is capable of delivering user-controlled variable suction, which allows for safer elevation of the floor of the third ventricle prior to fenestration, reducing the risk of BA injury. Images copyright Penumbra, Inc., used for academic purposes.

The endoscope was advanced to the foramen of Monro, which was entered to visualize the floor of the third ventricle (Figure 2B) including the mammillary bodies, optic chiasm, and infundibular recess. The Artemis neuro evacuation device (Figure 3) was then inserted into a side port of the neuroendoscope, after which the neuroendoscope and Artemis device were advanced to the midline, with the Artemis neuro evacuation device making contact with the floor of the third ventricle just anterior to the mammillary bodies (Figure 2C). Using gentle suction, the floor of the third ventricle was elevated to prevent inadvertent damage to the BA, and a fenestration was made in the tuber cinereum and membrane of Liliequist, exposing the apex of the BA just inferior to the membrane of Liliequist (Figure 2D). The neuroendoscope was advanced to the fenestration, and the BA was inspected and found to be undamaged (Figure 2E).

Pulsatile flow of CSF was observed through the fenestration in the tuber cinereum, and the sheath and neuroendoscope removed under visualization without significant bleeding. A piece of absorbable sponge was used to cover the cranial defect, the burr hole closed with a cranial fixation plate, and the incision closed.

Postoperative course

**Figure 4:**
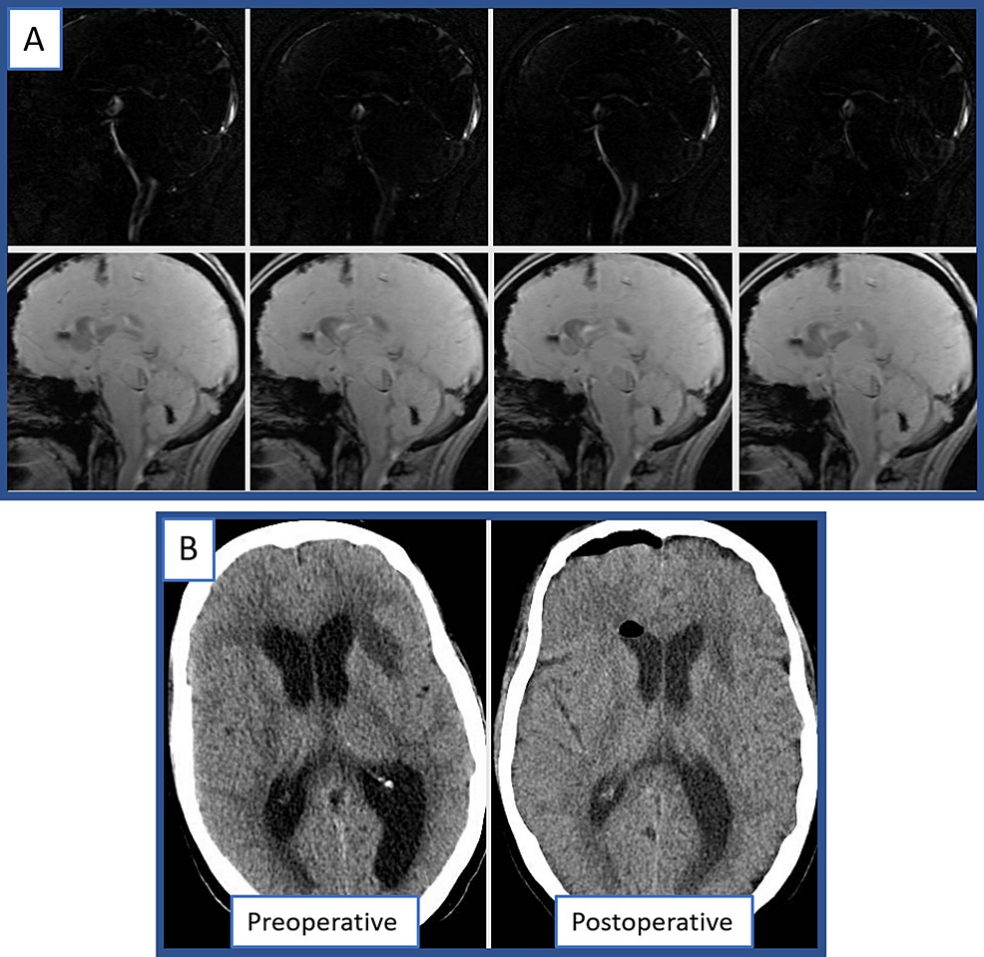
Postoperative imaging MRI flow study postoperatively demonstrated flow through the third ventricular floor fenestration (A), with a reduction in hydrocephalus visible on MRI as well as postoperative CT scans (B). This figure was prepared for this submission using original images, and no credit/license is required.

The patient tolerated the procedure without complication and was transferred to the post-anesthesia care unit. She returned to the neurosurgical intensive care unit where she had resolution of her preoperative subjective short-term memory loss and improvement in drift, ataxia and blurry vision. Her preoperative weakness and diplopia persisted. On postoperative day 1, a repeat MRI flow study (Figure 4A, Video 1) demonstrated CSF flow into the subarachnoid space with improvement in her obstructive hydrocephalus (Figure 4B) after which she was transferred to the neurosurgical floor. On postoperative day 3, she was discharged to a subacute rehabilitation facility at her neurologic baseline, after which she was able to continue SRS and immunotherapy with her primary oncologic team.

## Discussion

ETV represents an attractive treatment option for patients with hydrocephalus, especially in cases where the long-term success of CSF diversion is important, or where CSF shunting is not appropriate. While ETV has better long-term complication rates than VPS, it does carry a risk of short-term failure and complications, most notably damage to the BA [8]. During ETV, the fenestration of the third ventricular floor carries the highest risk for damage to the BA, and anatomic variants including a high riding BA increase this risk [7].

While the patient presented here had a high-riding BA, she also presented with obstructive hydrocephalus secondary to intracranial metastasis. To prevent neurologic complications including herniation, CSF diversion was necessary. Due to the desire to avoid the associated complications of VPS, as well as the theoretical risk of metastatic seeding, ETV was the preferred CSF diversion procedure. However, the presence of a high-riding BA complicated the clinical picture. Despite the challenges involved with ETV in the setting of abnormal BA anatomy, it remained the optimal intervention for her and thus required increased care and technical creativity to be performed safely.

The Artemis system consists of the neuro-evacuation device and variable-suction system (Figure 3). While first designed for evacuation of intracranial hematomas, Artemis has been used safely and effectively for a variety of other surgical scenarios including transsphenoidal resection of pituitary tumors [9], and removal of third ventricle colloid cysts [10]. While this is the first reported use of Artemis in the context of ETV, the techniques to perform ETV are now well established [11] and Artemis may be integrated into standard ETV procedures without major modifications to technique. 

It is the view of the authors that one advantage of Artemis in comparison to other methods of fenestrating the tuber cinereum and membrane of Liliequist is the ability to deliver a precise and controlled amount of suction during the fenestration process, allowing the operator to elevate the floor of the third ventricle. This increases the distance between the instrument and critical neurovascular structures and may allow the operator a greater degree of control over the fenestration, which is particularly important in the setting of a high-riding BA. Due to these properties, Artemis may allow for safer ETV even in the setting of a high-riding BA. It is possible that in the hands of a skilled operator, Artemis may be used to perform ETV in the setting of other abnormalities of the posterior circulation such as BA aneurysms.

## Conclusions

This case presents the first reported use of Artemis in ETV. Given the technical challenge of performing ETV in the setting of a high-riding BA, the characteristics of Artemis may make it a useful technical addition to ETV in such settings. In order to better characterize both the benefits as well as the limitations of using Artemis in ETV, further studies are required.
